# Change of Nurr1 expression in mouse hippocampal CA3 region following excitotoxic neuronal damage

**DOI:** 10.22038/IJBMS.2019.38712.9177

**Published:** 2020-02

**Authors:** Choong-Hyun Lee

**Affiliations:** 1 Department of Pharmacy, College of Pharmacy, Dankook University, Cheonan 31116, Republic of Korea

**Keywords:** Excitotoxic neuronal – damage, Hippocampal CA3 region, Kainic acid, Nurr1, Pyramidal cells

## Abstract

**Objective(s):**

Nuclear receptor-related protein 1 (Nurr1), one of immediate-early genes, is a member of orphan nuclear receptor family. The aim of this study was to investigate the time-dependent change of Nurr1 protein expression in the mouse hippocampal CA3 region following kainic acid (KA)-induced excitotoxic neuronal damage.

**Materials and Methods:**

Male ICR mice were used as experimental animals, and 30 mg/kg KA was administered intraperitoneally. To confirm the KA-induced neuronal damage in the hippocampal CA3 region, Fluoro-Jade B histofluorescence staining was performed. In addition, the time-dependent change of Nurr1 protein expression was also examined using immunohistochemistry and western blot analysis.

**Results:**

Marked neuronal damage was observed in the hippocampal CA3 region at 24 hr after KA injection. In addition, both Nurr1 immunoreactivity and protein level were significantly increased at 6 hr and 12 hr after KA injection, and then decreased at 24 hr after KA injection.

**Conclusion:**

This result indicates that KA-induced alteration of Nurr1 protein expression may be associated with the neuronal degeneration in the hippocampal CA3 region after KA injection.

## Introduction

Kainic acid (KA) has been used to produce an experimental animal model for study of human temporal lobe epilepsy (1-4). In addition, excitotoxicity by KA administration has been well known to result in the neuronal apoptosis and necrosis in some brain regions, including hippocampus, cerebral cortex and thalamus (4, 5). Especially, in the hippocampus, pyramidal neurons in the hippocampal CA3 region are well known as the most vulnerable area following KA-induced epilepsy, because of the highest abundance of kainate receptors (1-4, 6, 7). Some of the underlying molecular mechanisms related to KA-induced neuronal damage have been suggested, such as overactivation of glutamate receptor, increased calcium influx into neuron, excessive release of reactive oxygen species and proinflammatory cytokines (4, 8, 9).

It has been well known that immediate-early gene (IEG) participates in apoptotic cell death by modulation of gene transcription and that inhibition of the effects of IEG could attenuate apoptotic death (10, 11). In addition, IEGs, such as c-fos and c-jun, are upregulated during increased neuronal activity following seizure induction, and elevated IEGs expression leads to transcriptional activation of various genes, which modulate neuronal activity as well as vulnerability to neuronal damage (12, 13). Nuclear receptor-related protein 1 (Nurr1) is a member of orphan nuclear receptor family 4, and it is an IEG that is regulated by nuclear factor kappa B (14, 15). In the brain, Nurr1 is primarily expressed in dopaminergic neurons of midbrain, and plays essential roles in development and maintenance of dopaminergic neurons (16-18). On the other hand, Nurr1 is also expressed in other brain region including pyramidal neurons of hippocampus (19). Recent studies have reported that Nurr1 in hippocampus participates in hippocampal neurogenesis, cognitive function and stress (20, 21). 

Some previous studies have reported the change of Nurr1 expression in the rat or mouse hippocampus after KA injection (22-24). However, the relationships between changes of Nurr1 expression and excitotoxic neuronal damage in the hippocampus following epilepsy are not fully elucidated yet. Therefore, the objective of this study was to investigate the time-dependent change of Nurr1 protein expression in the mouse hippocampal CA3 region following KA-induced excitotoxic neuronal damage.

## Materials and Methods

***Experimental animals***

Eight-weeks old male ICR mice (body weight, 25.2 ± 1.9 g) were purchased from RaonBio Inc. (Yongin, South Korea). All experimental procedures were approved by Institutional Animal Care and Use Committee at Dankook University (approval number: DKU-15-038). All of the experiments were conducted to minimize the number of animals used and the suffering caused by the procedures used in the present study.

***KA injection***

KA (Sigma, MO, USA) was dissolved in sterile normal saline with concentration of 1 mg/ml, and 30 mg/kg KA was administered intraperitoneally. Dose of KA was selected based on the methods and results of the previous study (1). In case of death of experimental animals after KA injection, additional animals were added.

***Tissue processing***

Mice of control and pilocarpine-treated groups (n = 6 at each point in time) were sacrificed at designated times (6, 12 and 24 hr after KA injection). For the histological analysis, animals were anesthetized with zoletil 50 (60 mg/kg, Virbac, Carros, France) and perfused transcardially with 4% paraformaldehyde in 0.1 M phosphate-buffer. The brain tissues were removed and serially sectioned with a cryostat (Leica, Wetzlar, Germany) into 30-μm coronal sections.

***Fluoro-Jade B histofluorescence staining***

To confirm the KA-induced neuronal damage in the hippocampal CA3 region at 24 hr after KA injection, Fluoro-Jade B (F-J B) histofluorescence staining was performed according to the method of the previous studies (1, 3). In brief, the sections were first immersed in a solution containing 1 % sodium hydroxide in 80 % alcohol, and followed in 70 % alcohol. They were then transferred to a solution of 0.06 % potassium permanganate, and transferred to a 0.0004 % F-J B (Histochem, Jefferson, AR) staining solution. After washing, the sections were examined using an epifluorescent microscope (Carl Zeiss, Göttingen, Germany) with blue (450-490 nm) excitation light and a barrier filter.

***Immunohistochemistry for Nurr1***

According to the method of the previous studies (1-3), immunohistochemical staining for Nurr1 was performed using the hippocampal sections from experimental groups. In brief, the sections were incubated with rabbit anti-Nurr1 (1:100, Thermo Scientific, Waltham, MA, USA) and subsequently exposed to biotinylated goat anti-rabbit IgG and streptavidin peroxidase complex (1:200, Vector, Burlingame, CA). And, they were visualized by 3,3’-diaminobenzidine (Sigma, MO, USA).

To quantitatively analyze Nurr1 immunoreactivity, five sections per animal were selected with 180-μm interval, and digital images of the hippocampal CA3 region were observed and captured with Axio Imager 2 microscope (Carl Zeiss, Germany) equipped with a digital camera (Axiocam, Carl Zeiss). According to the method of a previous study (25), digital images were calibrated into an array of 512 X 512 pixels corresponding to a tissue area of 140 X 140 μm, and the mean immunoreactivity of Nurr1 in pyramidal neurons of the hippocampal CA3 region was measured by a 0-255 gray scale system. After the background density was subtracted, a ratio of the relative immunoreactivity (RI) of image file was calibrated as % using Adobe Photoshop version 8.0 and then analyzed using NIH Image 1.59 software. A ratio of the RI was calibrated as %, with the control-group designated as 100%. 

***Western blot analysis for Nurr1***

To examine changes in Nurr1 protein level in the hippocampus after KA treatment, western blot analysis for Nurr1 was performed according to the method of a previous study (26, 27). In brief, the animals in all experimental groups (n=5 at each point in time) were sacrificed at designated times (6, 12 and 24 hr after KA injection), and their brains were removed. The brains were then serially and transversely cut into 400 µm thickness sections using a vibratome (Leica Camera AG, Wetzlar, Germany). Subsequently, the hippocampal CA3 region was dissected with a surgical blade. The tissues were homogenized and centrifuged, and then the supernatants were used for western blot analysis. Rabbit anti-Nurr1 (1:150, Thermo Scientific) or mouse anti-β-actin (1:2,000, Sigma) was used as a primary antibody. After scanning the result of the western blot analysis, densitometric analysis of Western blot bands was performed using Image J 1.46 software (National Institutes of Health, Bethesda, MD, USA), to measure relative optical density (ROD). Ratios of the ROD of each experimental group were represented as %, with control-group designated as 100 %.

***Statistical analysis***

The data shown here represent the means±SEM. Differences of the means among the groups were statistically analyzed by analysis of variance (ANOVA) with a *post hoc* Bonferroni’s multiple comparison test. Statistical significance was considered at *P*<0.05.

## Results

***KA-induced mortality and neuronal damage in hippocampal CA3 region***

In the KA-group, 18/51 animals died within 6 hr after 30 mg/kg KA injection; therefore, KA-induced mortality was 35.3 % in this study. 

**Figure 1:**
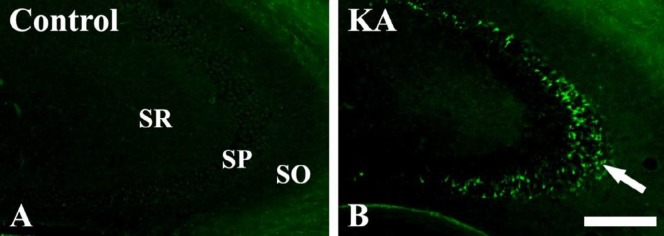
Fluoro-Jade B (F-J B) histofluorescence staining in the hippocampal CA3 region of the control- and kainic acid (KA)-groups at 24 hr after KA injection. Many F-J B positive cells (arrow) are detected in the stratum pyramidale (SP) of the CA3 region in the KA-group. SO: stratum oriens, SR: stratum radiatum. Scale Bar = 200 µm

In the control-group, no F-J B positive cells were observed in the hippocampal CA3 region (Figure 1A). However, F-J B positive cells were markedly increased in the stratum pyramidale (SP) of the KA-group at 24 hr after KA injection (Figure 1B).

***KA-induced alteration of Nurr1 immunoreactivity in hippocampal CA3 region***

**Figure 2:**
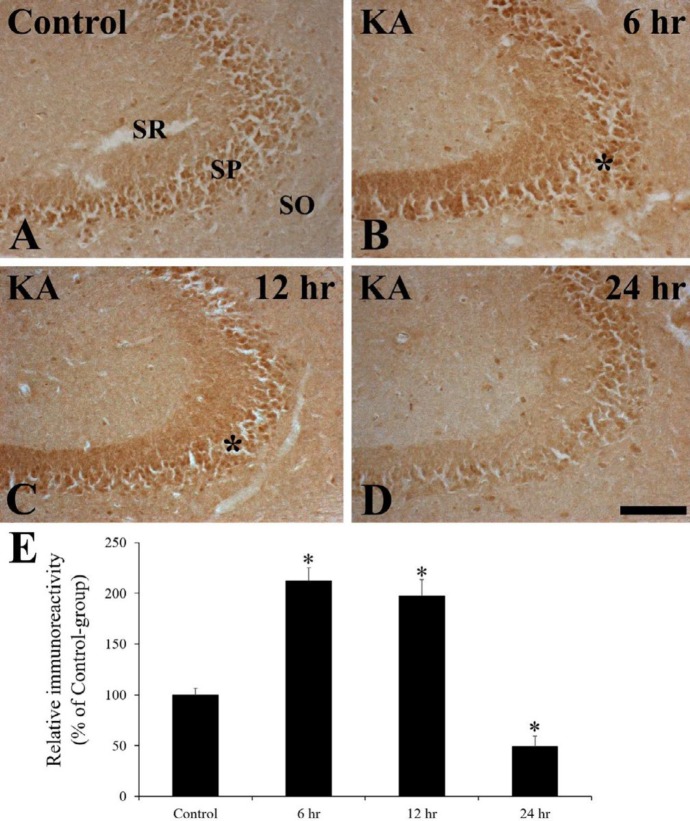
Nurr1 immunohistochemistry in the hippocampal CA3 region of the control- (A), kainic acid (KA)- (B-D) groups at 6 hr (B), 12 hr (C) and 24 hr (A, D) after KA injection. Nurr1 immunoreactivity is markedly increased at 6 hr and 12 hr after KA administration (asterisks), and then decreased in the hippocampal CA3 region of the KA-group. SO; stratum oriens, SP; stratum pyramidale, SR; stratum radiatum. Scale bar= 200 μm. E: Relative immunoreactivity (RI) as % of Nurr1 immunoreactivity in the pyramidal neurons of the hippocampal CA3 region in the control- and KA-groups (n=6 at each point in time, *P<0.05, significantly different from the control-group). Bars indicate the means±SEM

In the control-group, Nurr1 immunoreactivity was well observed in the pyramidal neurons of the SP in the hippocampal CA3 region (Figure 2A and 2E). However, in the KA-group, Nurr1 immunoreactivity was significantly increased about two times in the SP of the hippocampal CA3 region at 6 hr and 12 hr after KA injection (Figure 2B, 2C and 2E). And then, Nurr1 immunoreactivity was markedly decreased at 24 hr after KA injection, when KA-induced neuronal damage occurred (Figure 2D and 2E).

***KA-induced alteration of Nurr1 protein level***

**Figure 3:**
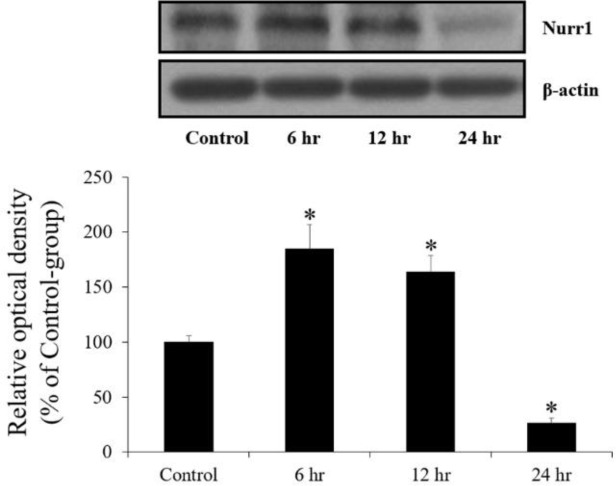
Western blot analysis of Nurr1 (~70 kDa) in the hippocampal CA3 region of the control- and kainic acid (KA)-groups at 6, 12 and 24 hr after KA injection (n=5 at each point in time, *P<0.05, significantly different from the control-group). Bars indicate the means±SEM

From western blot analysis, we observed that the KA-induced changes in Nurr1 protein level in the hippocampus were similar to those observed in the immunohistochemical data. Nurr1 protein level was significantly increased at 6 hr and 12 hr after KA injection and decreased at 24 hr after KA injection in the KA-group (Figure 3). 

## Discussion

It has been known that Nurr1 mRNA levels are significantly increased in the hippocampal CA3 region of adult rat from 1 hr to 16 hr after KA injection, and then return to basal level at 30 and 120 hr after seizure onset (22). Findings have shown that prolonged Nurr1 expression is associated with neuronal vulnerability to the neurotoxic effect of KA (22). It was also reported that Nurr1 mRNA expression was highly induced in pyramidal neurons of the rat hippocampal CA3 region at 30 min and 2 hr after KA administration, and then induction of Nurr1 was gradually decreased at 6 and 24 hr following seizure onset (23). Also, induction of IEG, such as Nurr1, and preceding apoptotic cell death after KA injection might indicate that vulnerable neurons could not reenter cell cycle and that might contribute to neuronal death observed after KA administration (23). In addition, Miller *et al*. (24) reported that a substantial increase in Nurr1 protein expression was observed in the hippocampal CA3 region of the mouse hippocampus at 24 hr after exposure to the threshold seizurogenic as well as the sub-threshold dose of KA. In this study, both Nurr1 immunoreactivity and protein level in the hippocampal CA3 region were significantly increased at 6 and 12 hr after KA injection. This result is in line with the results of above-mentioned previous studies, although there are some differences in animal models and dose of KA (22-24). On the other hand, it has also been known that down-regulated Nurr1 expression contributed to neuronal degeneration, and that restoration of Nurr1 expression could decrease apoptotic neuronal death in the substantia nigra of the MPTP (1- methyl-4- phenyl-1,2,3,6- tetrahydropyridine)-induced Parkinson’s disease mouse model (28). In the present study, Nurr1 protein level was also significantly decreased at 24 hr after KA injection compared to that of the control-group. Therefore, taken together, it is likely that both the transient increase and the marked reduction of Nurr1 protein expression might be related with the KA-induced neuronal degeneration in the hippocampal CA3 region. 

## Conclusion

This study showed that Nurr1 protein expression was apparently changed in the mouse hippocampal CA3 region after KA injection. This finding indicates that the marked alteration of Nurr1 protein expression may be associated with the KA-induced excitotoxic neuronal degeneration in the hippocampal CA3 region.
